# Performance of Omnipod Personalized Model Predictive Control Algorithm with Moderate Intensity Exercise in Adults with Type 1 Diabetes

**DOI:** 10.1089/dia.2019.0017

**Published:** 2019-05-07

**Authors:** Gregory P. Forlenza, Bruce A. Buckingham, Mark P. Christiansen, R. Paul Wadwa, Thomas A. Peyser, Joon Bok Lee, Jason O'Connor, Eyal Dassau, Lauren M. Huyett, Jennifer E. Layne, Trang T. Ly

**Affiliations:** ^1^ Barbara Davis Center for Diabetes, University of Colorado School of Medicine, Aurora, Colorado.; ^2^ Division of Pediatric Endocrinology, Department of Pediatrics, Stanford University, Stanford, California.; ^3^ Diablo Clinical Research, Walnut Creek, California.; ^4^ ModeAGC LLC, Palo Alto, California.; ^5^ Insulet Corporation, Acton, Massachusetts.; ^6^ Harvard John A. Paulson School of Engineering and Applied Sciences, Harvard University, Cambridge, Massachusetts.

**Keywords:** Artificial pancreas, Automated insulin delivery, Exercise, Closed-loop, Omnipod, Tubeless insulin pump

## Abstract

***Background:*** The objective of this study was to assess the safety and performance of the Omnipod^®^ personalized model predictive control (MPC) algorithm with variable glucose setpoints and moderate intensity exercise using an investigational device in adults with type 1 diabetes (T1D).

***Materials and Methods:*** A supervised 54-h hybrid closed-loop (HCL) study was conducted in a hotel setting after a 7-day outpatient standard treatment phase. Adults aged 18–65 years with T1D and HbA1c between 6.0% and 10.0% were eligible. Subjects completed two moderate intensity exercise sessions of >30 min duration on consecutive days: the first with the glucose set point increased from 130 to 150 mg/dL and the second with a temporary basal rate of 50%, both started 90 min pre-exercise. Primary endpoints were percentage time in hypoglycemia <70 mg/dL and hyperglycemia ≥250 mg/dL.

***Results:*** Twelve subjects participated in the study, with (mean ± standard deviation) age 36.5 ± 14.4 years, diabetes duration 21.7 ± 15.7 years, HbA1c 7.6% ± 1.1%, and total daily dose 0.60 ± 0.22 U/kg. Outcomes for the 54-h HCL period were mean glucose: 136 ± 14 mg/dL, percentage time <70 mg/dL: 1.4% ± 1.3%, 70–180 mg/dL: 85.1% ± 9.3%, and ≥250 mg/dL: 1.8% ± 2.4%. In the 12-h period after exercise start, percentage time <70 mg/dL was 1.4% ± 2.7% with the raised glucose set point and 1.6% ± 3.0% with reduced basal rate. The percentage time <70 mg/dL overnight was 0% ± 0% on both study nights.

***Conclusions:*** The Omnipod personalized MPC algorithm performed well and was safe during day and night use in response to variable glucose set points and with temporarily raised glucose set point or reduced basal rate 90 min in advance of moderate intensity exercise in adults with T1D.

## Introduction

Maintaining safe glycemic control during and after exercise is a challenge in managing type 1 diabetes (T1D). Exercise can provide many health benefits; however, fear of hypoglycemia and the loss of glycemic control may make participation in exercise difficult or daunting for people with T1D.^1–4^ Although there are extensive guidelines on the management of T1D during exercise, including recommended adjustments to insulin dosage and carbohydrate (CHO) consumption before, during, and after exercise,^1^ these guidelines can be challenging to implement in everyday life, and may not be adequate to prevent hypoglycemia during or after exercise.

Automated insulin delivery in response to continuous glucose monitor (CGM) signal has the potential to lower the barrier to exercise for people with T1D by improving glycemic outcomes and reducing the burden of management. Several studies have examined the performance of a single-hormone artificial pancreas (AP) system in response to exercise. The majority of these studies included exercise as part of the protocol without any announcement or adjustment to the algorithm.^5–14^ A few studies have included a user-initiated announcement of exercise^15–18^ or pre-exercise snacks,^19^ whereas others have included automatic detection of exercise through accelerometers or heart rate monitors.^20–23^ These studies have varied widely in the type of AP system, duration of use, duration and intensity of exercise, announcement strategy, amount and frequency of snacks, and outcomes reported, making it difficult to compare results directly or draw conclusions about which approach was most successful. However, in general, these studies have demonstrated that AP systems are able to maintain good glycemic control while reducing the occurrence of hypoglycemia, but supplemental CHO consumption is sometimes still necessary before, during, or after exercise to prevent or treat hypoglycemia.

The Omnipod Horizon™ Automated Glucose Control System is a single-hormone hybrid closed-loop (HCL) system using a personalized model predictive control (MPC) algorithm under development for commercial application.^24,25^ The objective of this study was to evaluate the safety and performance of the Omnipod personalized MPC algorithm in adults with T1D performing moderate intensity exercise in a supervised outpatient hotel setting. Although exercise safety was assessed using both glucose set point increase and basal rate reduction, the intent of this study was not to determine which method was superior but rather to test that both methods were safe and performed well.

## Materials and Methods

### Study design

**FIG. 1.**
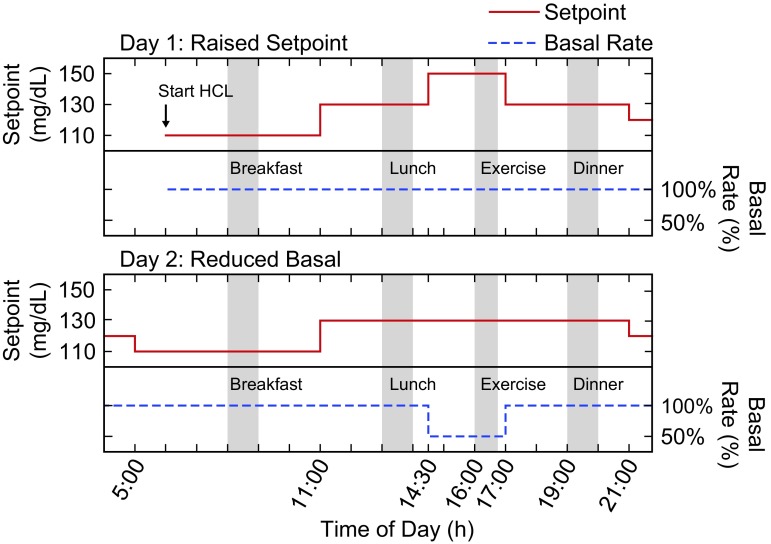
Representation of the algorithm input settings of glucose set point (mg/dL) and basal rate (% of usual rate) on each study day with exercise. On study day 1, the glucose set point was raised to 150 mg/dL 90 min before exercise start (approximate timing indicated by gray bar labeled Exercise), whereas the basal rate was maintained at 100% of the usual rate (top panel). On study day 2, the basal rate was decreased to 50% of the usual rate 90 min before exercise, whereas the glucose set point was maintained at 130 mg/dL (bottom panel). On both days, the glucose set point was set to 110 mg/dL in the early morning (05:00 h), increased to 130 mg/dL in the late morning (11:00 h), and lowered to 120 mg/dL in the late evening (21:00 h). The approximate meal times are indicated by gray bars labeled with meal type. On Study day 3, the glucose set point was set to 110 mg/dL in the early morning (05:00 h), and HCL ended ∼5 h after breakfast (not shown). HCL, hybrid closed loop.

This single-arm multicenter study assessed the Omnipod personalized MPC algorithm performance for 54 h with variable glucose set points in a supervised hotel setting, with the HCL period commencing before breakfast on day 1 and ending ∼5 h after breakfast on day 3 (Fig. 1). Participants engaged in a session of moderate intensity exercise lasting >30 min on each subsequent study afternoon at ∼1600 h, with example activities including soccer, basketball, and treadmill use. Exercise activities were similar on each day.

The algorithm was evaluated under two experimental conditions before exercise in a nonrandomized order: a temporary increase in the glucose set point or reduction of the preprogrammed subject-specific basal rate (Fig. 1). Ninety minutes before the first exercise session, the glucose set point was raised from 130 to 150 mg/dL, with the basal rate unchanged at 100% of the baseline rate. Ninety minutes before the second exercise session, a temporary basal rate reduction of 50% was implemented, with the glucose set point unchanged at 130 mg/dL. In both cases, the glucose set point and basal rate settings were returned to their original values at the end of exercise and dinner was consumed ∼2 h later. As a safety requirement, capillary blood glucose (BG) was required to be ≥120 mg/dL before commencing exercise. If capillary BG was <120 mg/dL, supplemental CHO was consumed without bolus until capillary BG was ≥120 mg/dL.

Throughout each study day, the glucose set point was changed according to the following schedule (in addition to the previously described changes made before exercise): start at 110 mg/dL in the early morning (05:00 h), increase to 130 mg/dL in the late morning (11:00 h), and lower to 120 mg/dL in the late evening (21:00 h) (Fig. 1). Subjects selected all meals from a variety of options containing 30–90 g CHO on the first day, with identical meals repeated on the second day for consistency between the 2 days with exercise sessions. Meal boluses were calculated by the system based on the amount of CHO estimated by the subject. This amount could be adjusted based on investigator judgment as needed. A correction or reverse bolus based on a recent capillary BG measurement could be given with the meal bolus at the discretion of the investigator or subject.

The HCL study was preceded by a 7-day outpatient standard treatment phase, during which subjects managed their diabetes at home per their usual routine using their personal insulin pump and a Dexcom G4 505 Share^®^ CGM (Dexcom, San Diego, CA). Pump settings were adjusted as needed by the investigator, based upon their clinical judgment.

### Study participants

The following were inclusion criteria for the study: age 18–65 years, T1D for ≥1 year, HbA1c >6% and ≤10% at screening, use of any insulin pump for ≥6 months, and total daily dose of insulin ≥0.3 U/kg. Subjects with ≥1 episode of severe hypoglycemia or diabetic ketoacidosis requiring an emergency room visit or hospitalization within the past 6 months, with hypoglycemic unawareness assessed by the Clarke Questionnaire,^26^ or who were pregnant or lactating were excluded. Each study site received Institutional Review Board approval and subjects provided written informed consent (Clinicaltrials.gov registration NCT03064906).

### Safety and monitoring

Study staff monitored subjects’ status throughout the HCL study period, with hypoglycemia (capillary BG <70 mg/dL or symptomatic) or severe hyperglycemia (capillary BG ≥300 mg/dL) treated per standard practice.^27^ HCL stopping criteria included BG ≥300 mg/dL and ketones ≥3.0 mmol/L, subjects unable to take oral CHO, loss of consciousness, seizure, or subject request.

### Investigational device

The investigational HCL system used in this study has been described previously.^24,25^ The system uses a modified version of the Omnipod^®^ Insulin Management System (Insulet Corp., Acton, MA) tubeless insulin pump (Pod) for insulin delivery, a modified personal diabetes manager (PDM), the Dexcom G4 505 Share AP System, and the Omnipod personalized MPC algorithm running on a Windows 10 tablet configured with the portable AP System.^28^ Operationally, the Dexcom CGM receiver communicated with the portable AP system (tablet) through a wired USB connection. The portable AP system transmitted insulin-dosing commands to the PDM through Bluetooth low energy relay. The PDM subsequently controlled insulin delivery by the Pod through a radiofrequency signal. The tablet was used to start each Pod, initiate HCL, display CGM and insulin delivery data, and for meal bolus delivery.^24,25^

Inputs to the investigational Omnipod personalized MPC algorithm included the subject-specific basal rate profile, total daily insulin dose, and the glucose set point. Correction factor and insulin-to-carbohydrate ratio are also entered into the system to be used for calculation of meal boluses and correction boluses. The Omnipod personalized MPC algorithm insulin-dosing decisions are made every 5 min based on CGM values to minimize the deviation between predicted BG for a 60-min horizon and the target glucose set point, while also minimizing deviations from the preprogrammed subject-specific basal rate.^24,25^ Temporary changes could be made to the subject-specific basal rate profile and glucose set point using the system interface. These parameters were adjusted during the study as described in Study Design (Fig. 1).

### Outcomes

The primary endpoints of this study were safety parameters of percentage of time the sensor glucose was in a hypoglycemic range, defined as <70 mg/dL, and hyperglycemic range, defined as ≥250 mg/dL, during the 54-h HCL study period with variable glucose set points. Secondary endpoints for the 54-h HCL study period included mean sensor glucose, percentage time with sensor glucose <54, <60, 70–140, 70–180, >180, and ≥300 mg/dL, and standard deviation (SD) and coefficient of variation (CV) of sensor glucose values.^29^ Additional endpoints were the immediate (2 h) and delayed (up to 24 h) sensor glucose response to moderate intensity exercise with a temporary raised glucose set point or reduced basal rate 90 min before exercise.

### Statistical analysis

As the primary endpoint for the study was safety, sample size was not determined by power calculation. Prespecified descriptive statistical analyses were performed for all subjects who entered the study (*n* = 12). Results were summarized for the 54-h HCL study period (overall) and the overnight period defined as 23:00 h to 07:00 h. Results were also summarized for the exercise sessions. Outcomes were calculated per subject and summarized as mean ± SD or median (interquartile range), unless otherwise indicated. Statistical analyses were performed using SAS^®^ 9.3 or later (SAS Institute, Cary, NC).

## Results

The characteristics of the 12 subjects are reported in Table 1. A summary of the glycemic measures and pump setting adjustments for the 7-day standard treatment run-in phase is included in the Supplementary Data (Supplementary Fig. S1 and Supplementary Table S1).

**Table 1.** Characteristics of the Study Population

*Characteristic*	*Subjects (*n* = 12)*
Age, years (range)	36.5 ± 14.4 (22.5–64.6)
Female, %	50
Diabetes duration, years (range)	21.7 ± 15.7 (4.2–51.7)
Insulin pump use duration, years (range)	11.4 ± 6.8 (3.3–28.2)
Insulin dose pre-HCL, U/(kg·d)^a^	0.60 ± 0.22
Insulin dose 24-h HCL, U/(kg·d)^b^	0.48 ± 0.11
HbA1c, %	7.6 ± 1.1^c^

Results are mean ± SD unless otherwise indicated.

^a^ Insulin dose averaged for the 7-day standard therapy run-in phase.

^b^ Insulin dose during entire HCL study period.

^c^ One subject with an HbA1c of 5.6% was allowed to enroll based on investigator discretion.

HCL, hybrid closed loop; SD, standard deviation.

### Glycemic outcomes

The glycemic outcomes for the 54-h HCL study period with variable glucose set points overall, during daytime (07:00–23:00 h), and overnight (23:00–07:00 h) are given in Table 2. The percentage of time with sensor glucose in the hypoglycemic range of <70 mg/dL was mean ± SD: 1.4% ± 1.3% during the 54-h HCL period overall and 0.0% ± 0.0% overnight. The percentage of time with sensor glucose in the hyperglycemic range of ≥250 mg/dL was 1.8% ± 2.4% for the overall HCL period and 0.1% ± 0.3% overnight. The percentage of time with sensor glucose in the target range of 70 to 180 mg/dL was 85.1% ± 9.3% overall and 93.4% ± 14.2% overnight. The mean glucose was 136 ± 14 mg/dL overall and 129 ± 23 mg/dL overnight.

**Table 2.** Glycemic Outcomes During the 54-h Hybrid Closed-Loop Period

*Parameter*	*Overall (54 h)*	*Day (07:00–23:00 h)*	*Night (23:00–7:00 h)*
Mean sensor glucose, mg/dL	136 ± 14	139 ± 13	129 ± 23
Standard deviation, mg/dL	38.5 ± 8.6	42.6 ± 10.9	19.6 ± 6.3
Coefficient of variation, %	28.2 ± 5.4	30.4 ± 6.8	15.5 ± 5.2
Percentage time in glucose range, %			
<54 mg/dL	0.2 ± 0.3	0.2 ± 0.5	0.0 ± 0.0
	0.0 (0.0–0.1)	0.0 (0.0–0.1)	0.0 (0.0–0.0)
<60 mg/dL	0.5 ± 0.6	0.7 ± 0.9	0.0 ± 0.0
	0.1 (0.0–0.8)	0.1 (0.0–1.2)	0.0 (0.0–0.0)
<70 mg/dL	1.4 ± 1.3	2.1 ± 1.9	0.0 ± 0.0
	1.7 (0.0–2.4)	2.4 (0.0–3.5)	0.0 (0.0–0.0)
70–140 mg/dL	60.3 ± 16.5	56.1 ± 13.2	69.4 ± 32.7
70–180 mg/dL	85.1 ± 9.3	81.3 ± 8.7	93.4 ± 14.2
>180 mg/dL	13.5 ± 9.5	16.6 ± 8.6	6.6 ± 14.2
≥250 mg/dL	1.8 ± 2.4	2.5 ± 3.3	0.1 ± 0.3
≥300 mg/dL	0.5 ± 1.0	0.8 ± 1.5	0.0 ± 0.0

Results are sensor glucose values, mean ± SD or median (IQR); SI conversion factor to convert glucose to mmol/L, multiply by 0.0555.

IQR, interquartile range.

### Exercise challenge

**FIG. 2.**
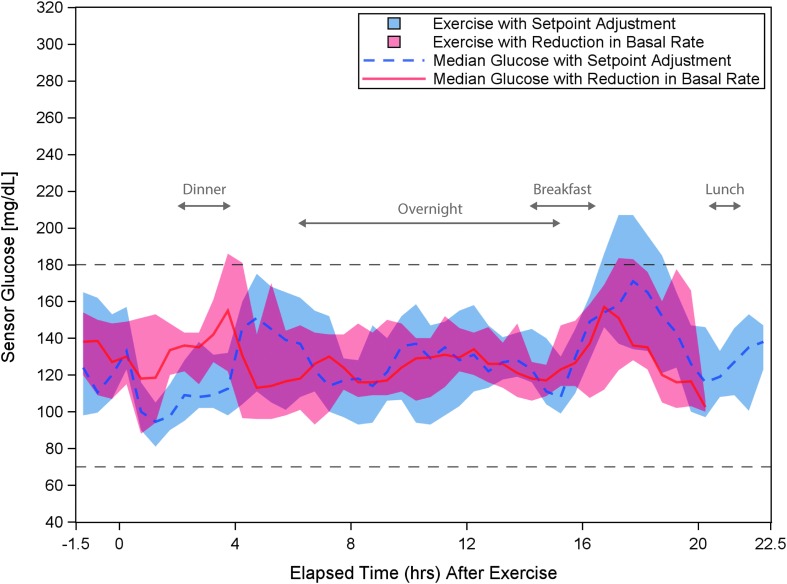
Comparison of the glycemic response for the 12 participants for the 24 h period beginning 90 min before exercise start with glucose set point increase or reduction in basal rate. Median sensor glucose response is plotted for the 12 participants for 24 h after algorithm glucose set point increase (dashed blue line) or basal rate decrease (solid red line). The shaded area represents the IQR. Exercise start times ranged from 15:43 h to 16:48 h. The approximate ranges of time for dinner, the overnight period, breakfast, and lunch are labeled on the graph. The study ended ∼20 h after the start of exercise on day 2 and, therefore, the basal rate adjustment data series is limited to 20 h on the graph. The target range of 70–180 mg/dL is indicated by black dashed lines. IQR, interquartile range.

Exercise duration was 39 ± 8 min and 38 ± 6 min in the raised glucose set point and reduced basal rate conditions, respectively. The 24-h glycemic response to the moderate intensity exercise on each study day is shown in Figure 2. Glycemic outcomes for the 2 and 12 h periods from exercise start are given in Tables 3 and 4. Glycemic outcomes were similar with the raised glucose set point and reduced basal rate pre-exercise. Hypoglycemia treatments and supplemental CHO ingestion in the time periods before exercise through the subsequent overnight period are summarized in Table 5. There were no supplemental CHO or hypoglycemic episodes (capillary BG <70 mg/dL) overnight in either group.

**Table 3.** Glycemic Outcomes Pre-exercise and for the Short-Term (2 h) Response to Exercise

*Mean glucose, mg/dL*	*Raised set point*	*Reduced basal*
Pre-exercise	140 ± 59	136 ± 26
90 min period before exercise start		
During exercise	140 ± 32	132 ± 26
Period from exercise start to end		
Postexercise	100 ± 19	122 ± 35
60 min period after exercise end		
During and postexercise	113 ± 17	128 ± 26
2 h period from exercise start		

Results are sensor glucose values, mean ± SD; SI conversion factor to convert glucose to mmol/L, multiply by 0.0555.

**Table 4.** Glycemic Outcomes for the Extended (12 h) Response to Exercise

	*Raised set point*	*Reduced basal*
Mean glucose, mg/dL	127 ± 24	131 ± 18
Percentage time in glucose range, %		
<54 mg/dL	0.7 ± 1.5	0 ± 0
	0.0 (0.0–0.3)	0.0 (0.0–0.0)
<70 mg/dL	1.4 ± 2.7	1.6 ± 3.0
	0.0 (0.0–1.7)	0.0 (0.0–2.1)
70–180 mg/dL	88.9 ± 17.6	89.1 ± 11.3
>180 mg/dL	9.7 ± 18.1	9.2 ± 11.8
≥250 mg/dL	0.9 ± 3.2	0.4 ± 1.0

Results are sensor glucose values, mean ± SD, or median (IQR); 12 h period measured from the start of exercise; SI conversion factor to convert glucose to mmol/L, multiply by 0.0555.

**Table 5.** Supplemental Carbohydrates and Hypoglycemia Around Exercise

	*Raised set point*			*Reduced basal*		
*Time period*	*Subjects with supplemental CHO,* n^a^	*Subjects with BG <70 mg/dL,* n	*CHO per subject (range, g)*	*Subjects with supplemental CHO,* n^a^	*Subjects with BG <70 mg/dL,* n	*CHO per subject (range, g)*
Before exercise (90 min)	5	1	25–40	5	0	16–27
During exercise (∼40 min)	1	0	12	0	0	—
Exercise end to dinner	4	2	12–20	3	1	14–17
Dinner to overnight	2	1	32–38	1	0	17
Overnight (23:00–07:00 h)	0	0	—	0	0	—

^a^ Supplemental CHOs are defined as CHO ingested without a corresponding insulin bolus. Includes participants consuming supplemental CHOs as treatment for capillary BG <70 mg/dL, as well as for other reasons such as participant request or to qualify for exercise with capillary BG >120 mg/dL. For supplemental CHO consumption not associated with a capillary BG <70 mg/dL, subjects were counted if they consumed at least 12 g of CHO within a period of 15 min or less.

BG, blood glucose; CHOs, carbohydrates.

### Safety outcomes

There were no serious adverse events, and the full HCL period was completed for all subjects with no instances of the stopping criteria being met. In the 648 patient-hours of HCL use, there were 0 hyperglycemic events involving capillary BG values ≥300 mg/dL. There were 13 hypoglycemic events in 8 subjects involving capillary BG values <70 mg/dL, resulting in 15 oral CHO treatments given (8–21 g CHO). This is equivalent to 0.48 events per subject per day overall.

### Percentage time in HCL

The mean percentage of the total HCL study period spent with the system running in closed loop was 98.8% ± 1.8% (range: 93.7%–100.0%). There were no suspected infusion site failures during the HCL period. The causes for interruption of closed loop included Pod replacement, temporary loss of CGM communication, or loss of system battery charge.

## Discussion

This multicenter feasibility study demonstrated that the Omnipod personalized MPC algorithm performed well and was safe during day and night use for 54 h in adults with T1D performing moderate intensity exercise with a temporary glucose set point increase or basal rate reduction 90 min before exercise. With each pre-exercise announcement strategy, the algorithm was able to attenuate insulin delivery to reduce the risk of immediate and delayed exercise-related hypoglycemia, with no instances of overnight hypoglycemia. Elimination of nocturnal hypoglycemia during this study period suggests that the combination of pre-exercise announcement with the Omnipod personalized MPC algorithm may be protective against overnight hypoglycemia after exercise. Importantly, there was no rebound hyperglycemia overnight after exercise. In addition, the system was safe when used with variable glucose set points throughout the day. To our knowledge, this was the first study to evaluate both the immediate and overnight responses to exercise during HCL when using an announcement strategy to reduce insulin delivery 90 min pre-exercise. Similar to approaches used to manage insulin delivery with exercise when using an insulin pump with standard therapy, these announcement strategies will require user engagement and may not be realistic for all situations; however, we have evaluated and found to be safe two simple approaches intended to reduce or prevent both immediate and delayed hypoglycemia after exercise during HCL.

The primary concern after exercise in patients with T1D is the risk of immediate or delayed hypoglycemia, especially overnight.^1,30–34^ Clinical evidence shows that reducing or suspending insulin at the start of exercise may not be adequate to prevent hypoglycemia,^35,36^ and that reducing insulin delivery 90 min before the start of exercise may be the best approach to attenuate hypoglycemia with exercise.^36,37^ In this study, we evaluated two options for an exercise announcement strategy to reduce insulin delivery 90 min before the start of exercise during HCL: raising the algorithm glucose set point or reducing the basal rate. The rationale behind each of these announcement strategies was to reduce the amount of insulin on board and bring the BG to a suitable level for the start of exercise, while allowing the algorithm to respond to the variation in insulin requirements that occurs post-exercise.

There was no hypoglycemia observed during the overnight periods after exercise. The absence of overnight hypoglycemia is promising compared with several recent studies of exercise using single-hormone HCL systems, whether the exercise was unannounced, announced at onset, or automatically detected, which have reported between 0.17% and 3% average time <70 mg/dL overnight.^9,13,17,23^ A study of a dual-hormone system with automatic exercise detection also showed 0.6% of time overnight <70 mg/dL.^23^

Although there was no hypoglycemia overnight, a small number of subjects experienced hypoglycemia in the short-term period after exercise. Three subjects required treatment with supplemental CHO for capillary BG <70 mg/dL after exercise with the glucose set point increase, and one subject with the basal rate reduction, all occurring within 3 h of exercise end. This result is consistent with previous studies, where use of a single- or dual-hormone HCL system with an announcement strategy in advance of exercise start was not able to prevent hypoglycemia during or shortly after exercise.^15,16^ For example, Jayawardene et al.^15^ evaluated a single-hormone HCL system in adults with T1D performing 45 min of moderate intensity exercise, with the glucose set point raised from 120 mg/dL to 150 or 160 mg/dL 2 h pre-exercise. Despite the early glucose set point change, one subject experienced hypoglycemia immediately after exercise. In Taleb et al.,^16^ adults with T1D completed 60 min of aerobic exercise while using a single- or dual-hormone HCL system, with the glucose set point raised from 95 to 150 mg/dL 20 min before exercise start. Nine and three subjects experienced hypoglycemic events with BG <70 mg/dL after exercise with the single- and dual-hormone systems, respectively. The set point adjustment made 20 min pre-exercise may not have allowed a sufficient decrease in insulin on board, as compared with the recommended 90 min of reduced insulin delivery before exercise.^1^

A potential concern when reducing insulin delivery before exercise is the increased risk of delayed hyperglycemia; however, the exercise announcement strategies used in this study were not associated with increased hyperglycemia post-exercise. The percentages of time with CGM >180 and ≥250 mg/dL in the 12 h after exercise and overnight remained low and were consistent with overnight results previously reported for the Omnipod personalized MPC algorithm in adults not performing exercise^25^ (no data are available for direct comparison for 12 h post-exercise).

A review of the literature^38^ indicates that HCL systems may be expected to achieve at least 70% of sensor glucose values between 70 and 180 mg/dL, <4% of values <70 mg/dL, a CV <36%,^39^ and a mean glucose of ≤155 mg/dL, equivalent to an estimated HbA1c of 7.0%.^40,41^ This study exceeded each of these overall glycemic control performance metrics, with 85.1% of sensor glucose values in the target range of 70–180 mg/dL overall and an average CV of 28.2%. In addition, the results compare favorably with other recent studies of HCL in adults with T1D participating in exercise, which have shown mean percentages of time in target range between 64% and 88%.^9,13,17,22,23^ The glycemic control metrics were exceeded in this study even in the presence of daily moderate intensity exercise sessions and with variable algorithm glucose set points throughout each day. These results provide the first demonstration of safe use of variable set points with the Omnipod personalized MPC algorithm.

The limitations of this study include the absence of a standard care control arm; however, previous studies have shown superior hypoglycemia results using HCL with exercise as compared with standard care.^7,23^ In addition, exercise sessions were not standardized on subsequent days for intensity, duration, or pre-exercise supplemental CHO consumption, so it is not possible to directly compare the two announcement strategies. Only one type of exercise was studied and, therefore, the results may not apply to other types of exercise, such as high-intensity interval training or extended periods of intense aerobic exercise.^1^ Lastly, the study had a relatively short duration of HCL conducted in a supervised hotel setting. Additional challenges to the algorithm may be faced in an unsupervised environment or when the system is used for longer periods of time.

## Conclusions

This feasibility study demonstrated that the Omnipod personalized MPC algorithm performed well and was safe for 54 h of use by adults in the outpatient hotel setting. In addition, the system was able to maintain good glycemic control within target ranges in the presence of glucose set point changes and moderate intensity exercise. The exercise announcement strategies of either a temporarily raised glucose set point or reduced basal rate 90 min before exercise were found to be safe methods to prepare for exercise during HCL, with some subjects consuming supplemental CHO before or after exercise. No subject experienced hypoglycemia on either night after exercise. Longer term outpatient studies will assess safety and performance of the algorithm during extended use under free-living conditions in patients of all ages with T1D.

## Supplementary Material

Supplemental data

Supplemental data

Supplemental data
