# Supplementary material for: Performance of Omnipod Personalized Model Predictive Control Algorithm with Moderate Intensity Exercise in Adults with Type 1 Diabetes
Source: Diabetes Technol Ther. 2019 May 7;21(5):265–72. doi: 10.1089/dia.2019.0017 (PMC6532546; doi:10.1089/dia.2019.0017)
Supplement: Supplemental data [file Supp_Data.pdf]

Supplementary Data

Changes to Pump Parameters During Standard Therapy Run-In Phase

The glycemic measures from the 7-day standard treatment phase are given in Table 2. In the standard therapy phase, seven (58%) subjects had adjustments to their basal rate with increases made for four subjects and decreases made for three subjects. Changes were made to the insulin-to-carbohydrate ratios for six subjects (50%) with increases (decreased insulin delivery) in one subject and decreases (increased insulin delivery) in five subjects. Correction factors were adjusted for three (25%) subjects with increases (decreased insulin delivery) in one subject and decreases (increased insulin delivery) in two subjects. One subject did not have insulin-to-carbohydrate or correction factor settings before the standard therapy phase.
