# Supplementary material for: Performance of Omnipod Personalized Model Predictive Control Algorithm with Moderate Intensity Exercise in Adults with Type 1 Diabetes
Source: Diabetes Technol Ther. 2019 May 7;21(5):265–72. doi: 10.1089/dia.2019.0017 (PMC6532546; doi:10.1089/dia.2019.0017)
Supplement: Supplemental data [file Supp_Fig1.pdf]

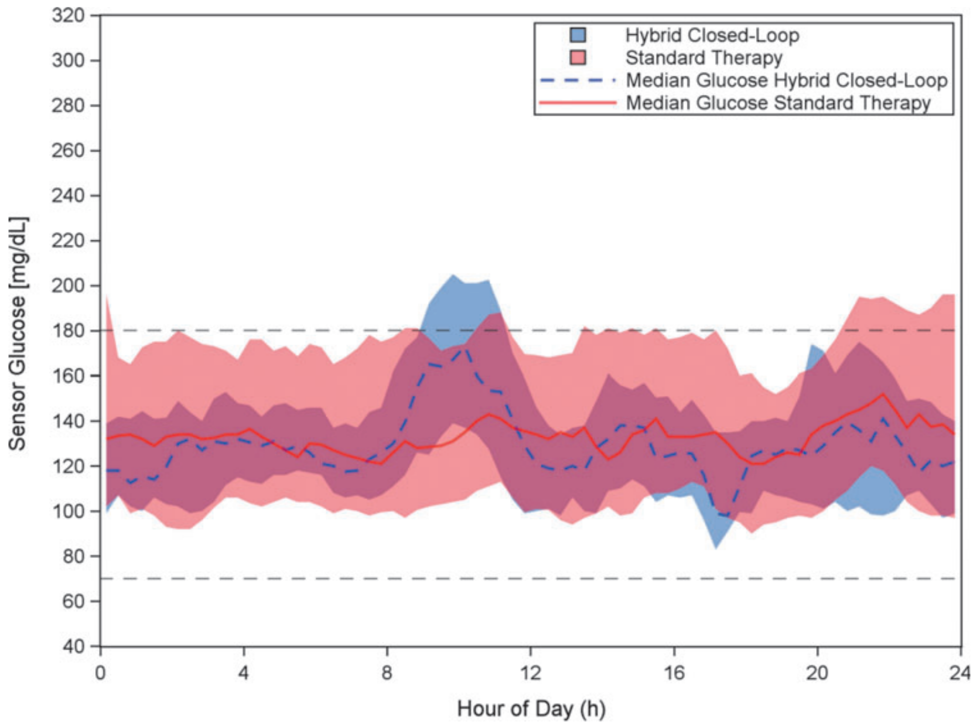


**SUPPLEMENTARY FIG. S1.** Sensor glucose versus time of day for 12 adult subjects during 54 h of hybrid closed-loop control (blue), with data from 1 week of standard therapy shown as comparison (red). The data are presented as median (line) and interquartile range (shaded area) of sensor glucose per time of day across all subjects and days. The target range of 70–180 mg/dL is indicated by black dashed lines.
