# Supplementary material for: Performance of Omnipod Personalized Model Predictive Control Algorithm with Moderate Intensity Exercise in Adults with Type 1 Diabetes
Source: Diabetes Technol Ther. 2019 May 7;21(5):265–72. doi: 10.1089/dia.2019.0017 (PMC6532546; doi:10.1089/dia.2019.0017)
Supplement: Supplemental data [file Supp_Table1.pdf]

Supplementary Table S1. Glycemic Outcomes During the 7-Day Standard Therapy Phase

Parameter	*Overall (7-day)*	*Night (23:00–7:00)*
Mean sensor glucose, mg/dL	143 ± 18	144 ± 20
Standard deviation, mg/dL	52.1 ± 11.7	54.2 ± 13.5
Coefficient of variation, %	36.1 ± 5.7	37.4 ± 6.8
Percentage time in glucose range, %		
<54 mg/dL	1.1 ± 1.3	1.7 ± 2.1
	0.7 (0.4–1.2)	1.1 (0.0–2.1)
<70 mg/dL	4.6 ± 3.1	6.0 ± 4.4
	4.3 (2.9–5.1)	6.1 (2.6–8.2)
70–180 mg/dL	73.0 ± 14.8	71.1 ± 16.7
>180 mg/dL	22.5 ± 14.1	22.9 ± 16.0
≥250 mg/dL	5.5 ± 4.9	7.0 ± 6.4
